# Molecular mechanisms of osteosarcoma metastasis and possible treatment opportunities

**DOI:** 10.3389/fonc.2023.1117867

**Published:** 2023-05-01

**Authors:** Xinhui Du, Hua Wei, Boya Zhang, Bangmin Wang, Zhehuang Li, Lon Kai Pang, Ruiying Zhao, Weitao Yao

**Affiliations:** ^1^ Bone Soft Tissue Department, The Affiliated Cancer Hospital of Zhengzhou University and Henan Cancer Hospital, Zhengzhou, China; ^2^ Key Laboratory for Digital Assessment of Spinal-Pelvic Tumor and Surgical Aid Tools Design (Zhengzhou), Zhengzhou, Henan, China; ^3^ Key Laboratory for Perioperative Digital Assessment of Bone Tumors (Henan), Zhengzhou, Henan, China; ^4^ Department of Anesthesiology, Pain and Perioperative Medicine, The First Affiliated Hospital of Zhengzhou University, Zhengzhou, Henan, China; ^5^ Baylor College of Medicine, Houston, TX, United States; ^6^ Department of Integrative Biology and Pharmacology, McGovern Medical School, The University of Texas Health Science Center at Houston, Houston, TX, United States

**Keywords:** osteosarcoma, metastasis, reprogramming, tumor microenvironment, mechanism

## Abstract

In osteosarcoma patients, metastasis of the primary cancer is the leading cause of death. At present, management options to prevent metastasis are limited and non-curative. In this study, we review the current state of knowledge on the molecular mechanisms of metastasis and discuss promising new therapies to combat osteosarcoma metastasis. Genomic and epigenomic changes, metabolic reprogramming, transcription factors, dysregulation of physiologic pathways, and alterations to the tumor microenvironment are some of the changes reportedly involved in the regulation of osteosarcoma metastasis. Key factors within the tumor microenvironment include infiltrating lymphocytes, macrophages, cancer-associated fibroblasts, platelets, and extracellular components such as vesicles, proteins, and other secreted molecules. We conclude by discussing potential osteosarcoma-limiting agents and their clinical studies.

## Introduction

Osteosarcoma is the most common primary malignant bone tumor in children and young adults. Current treatment options for osteosarcoma include neoadjuvant chemotherapy, wide tumor resection, and adjuvant chemotherapy. Unfortunately, these treatment options are limited in efficacy, and management outcomes have not improved in the last 30 years. The 5-year overall survival of osteosarcoma patients with primary localized tumors is 60%–70%, whereas survival drops to approximately 20% in patients with metastasis (1). Distant metastasis is found in approximately 10% of patients at diagnosis, but eventually develops in approximately 50% of patients, commonly contributing to death (2). Hence, one approach to improving overall survival in patients with osteosarcoma is to prevent or delay tumor metastasis. While the mechanisms governing osteosarcoma metastasis remain unclear, developments in molecular technology have enabled us to study osteosarcoma and other cancers more closely. These findings help to pave the way towards novel, effective, and hopefully curative therapies.

**Figure 1:**
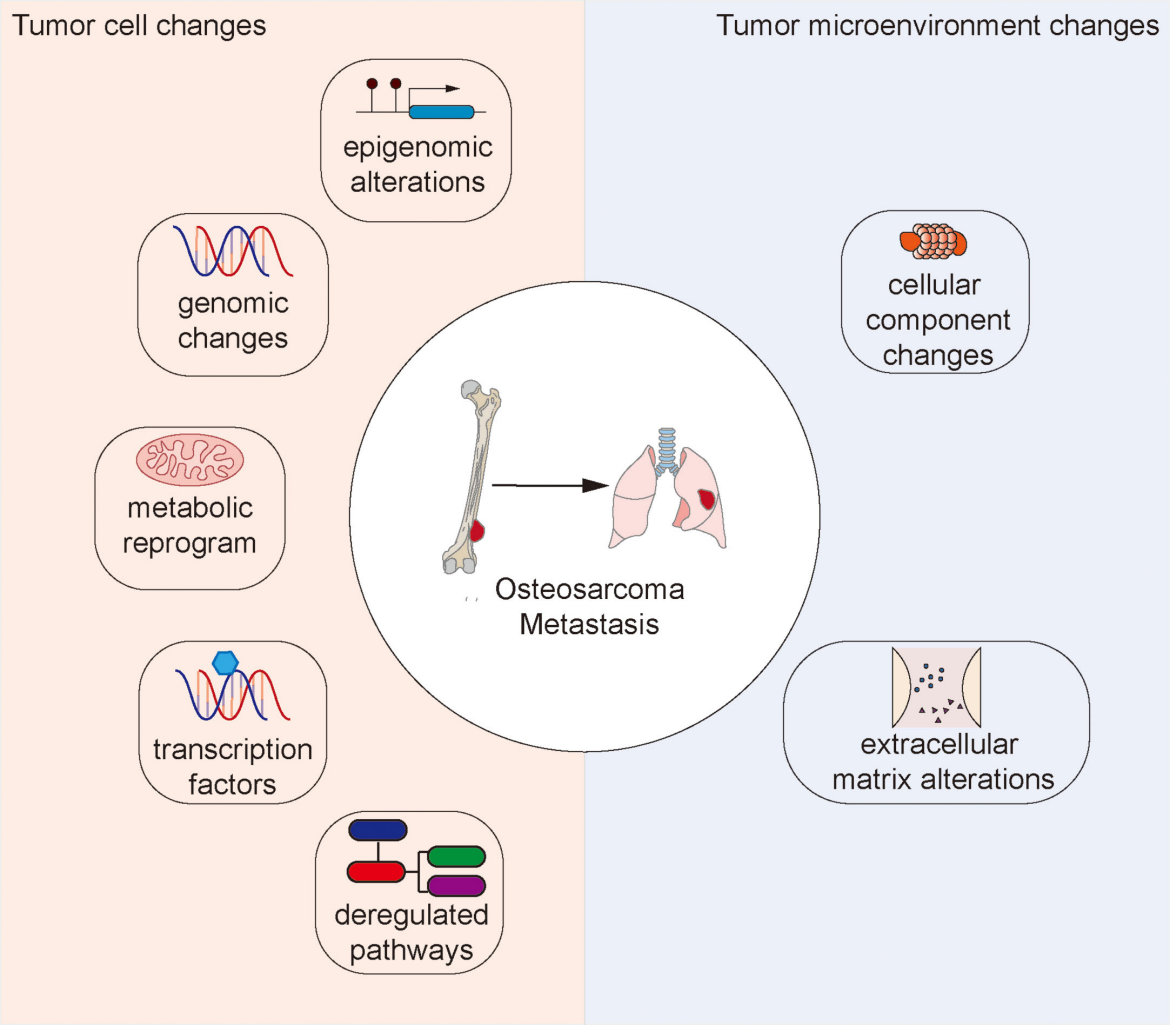
Changes to the tumor cell and tumor microenvironment that facilitate osteosarcoma metastasis.

In this review, we discuss recent studies that highlight potential factors implicated in osteosarcoma metastasis (**Figure 1**), and highlight a few emerging anti-cancer agents with potential anti-metastatic activity.

### Tumor cell alterations

#### Genomic alterations

The genomic profile of osteosarcoma differs greatly from that of other malignant tumors. For example, unlike in breast cancer or melanoma, few targetable recurrent point mutations exist within the protein-coding genes identified in osteosarcoma. In addition, widespread recurrent somatic copy number alterations (SCNAs) and structural rearrangements have been detected and proposed to be responsible for osteosarcoma carcinogenesis and progression. Even among osteosarcoma patients, SCNAs and structural rearrangements are highly heterogeneous (3).

Among osteosarcoma samples, metastatic tumors demonstrate significantly higher mutational burden and genomic instability than primary tumors. Mutated genes are enriched in the PI3K-Akt pathway at both the early and late stages of tumor evolution and in the MAPK pathway at the metastatic stage (4).

Examination of metastatic samples of osteosarcoma revealed alterations in key genes that may play vital roles in metastasis. These alterations include the loss of TP53, RB1, and CDKN2A, or the gain of MYC and MDM2 (4). TP53 is commonly mutated in various cancers including osteosarcoma, and most of the mutations occur in the DNA-binding domain and are characterized as either structural or contact mutations. In addition to inhibitory effects on wild-type TP53 activity, gain-of-function activity promoting tumor progression was also noted. Studies have shown that contact mutations are stronger drivers of osteosarcoma metastasis (5).

RB1 is a well-established tumor suppressor gene reported to be mutated in multiple malignant tumor types including osteosarcoma. RB1 mutation in osteosarcoma is responsible for tumor carcinogenesis and progression. At a molecular level, RB1 loss leads to aberrant spliceosome function due to the upregulation of E2F3a, a mediator of spliceosome gene expression (6).

Amplification of 17p11.2 chromosomal region containing TOP3A led to increased expression of TOP3A, which supported the maintenance of telomeres through the alternative lengthening of telomeres (ALT) mechanism in osteosarcoma (3).

Structural rearrangements in osteosarcoma can also result in novel fusion genes that may participate in tumor progression and metastasis. For example, the fusion gene Rab22a-NeoF1 was detected in osteosarcoma samples. The resultant fusion protein activates RhoA and promotes cell migration, invasion, and lung metastasis after acetylation on K7 (7). When secreted, it also alters the function of adjacent tumor-negative cells and stimulates macrophages toward M2 polarization (8).

Personalized therapy targeting patient-specific genes with copy number alterations and expression changes was tested in patient-derived tumor xenografts and showed a significant decrease in tumor burden (9).

#### Epigenomic changes

Epigenetic changes are commonly found in osteosarcoma and are involved in multiple aspects of tumor progression including metastasis (10). For example, the methyltransferase DNMT3A inhibits miR-149 expression by DNA methylation to activate the NOTCH1/Hedgehog pathway, thereby promoting the proliferation and metastasis of osteosarcoma (11). The long non-coding RNA (lncRNA) THAP9-AS1 binds to and promotes methylation of the SOCS3 promoter region with DNA methyltransferases (DNMTs) and activates the JAK2/STAT3 signaling pathway to facilitate osteosarcoma growth and metastasis (12). In fact, inhibiting DNMT-1 sensitized osteosarcoma cells to cabozantinib and other targeted agents by repressing the Notch pathway and subsequently upregulating expression of miR-34a (13).

RNA modifications also play a role in osteosarcoma metastasis. The m6A demethylase FTO mediates mRNA demethylation, promoting the decay of KLF3 mRNA and decreasing its expression, consequently facilitating osteosarcoma proliferation and metastasis (14). Also, the destabilizing effects of FTO on DACT1 mRNA promotes Wnt signaling and consequently osteosarcoma metastasis (15). In addition, ALKBH5-mediated m6A methylation upregulates the expression of USP22 and RNF40, subsequently inhibiting the ubiquitination of histone H2A, promoting osteosarcoma growth and metastasis (16). Upregulation of TRIM7 due to the loss of m6A RNA modifications has also been reported to promote osteosarcoma metastasis and chemoresistance by inducing the ubiquitination of BRMS1 (17).

The prognostic role of epigenetic changes in osteosarcoma have also been extensively studied. Immune-related DNA methylation patterns can be used to predict survival and tumor microenvironment patterns (18). RNA methylation-related signatures of metabolic genes and lncRNAs have also been proposed to be useful tools in the estimation of patient survival and immune landscapes of osteosarcoma (19, 20).

#### Metabolic reprogramming

Metabolic reprograming is one of the key features of osteosarcoma, and its role in tumor progression, drug resistance, and metastasis is well established (21). Various metabolic gene signatures have been found to predict survival in osteosarcoma patients (19, 22–24). For example, comprehensive metabolic profiling of osteosarcoma based on UHPLC-HRMS unveiled a panel of two metabolites, 5-aminopentanamide and 13(S)-HpOTrE (FA 18:3 + 2O), which was found to be an accurate indicator of lung metastases (25).

Aerobic glycolysis, also known as the Warburg effect, supports biosynthesis and metabolic processes necessary for osteosarcoma growth and metastasis (26). Key enzymes involved in this process, such as PGC1α, PKM2, ALDOA, and LDHA, can directly influence tumor progression and metastasis. For instance, miR-23b-3p downregulates PGC1α and promotes a metabolic shift from oxidative phosphorylation to glycolysis, supporting osteosarcoma progression (27).

PKM2 is another key enzyme regulating glycolysis, which acts on its substrate phosphoenolpyruvate (PEP) to form pyruvate (28). IRF7 was found to downregulate PKM2 *via* transcriptional suppression, inhibiting aerobic glycolysis in osteosarcoma (29). The SLIT2/ROBO1 axis contributes to the Warburg effect by activating the SRC/ERK/c-MYC/PFKFB2 pathway in osteosarcoma (30). ROCK2 can promote glycolysis and osteosarcoma tumor growth by upregulating HKII *via* the pPI3K/AKT signaling pathway (31). Aldolase A (ALDOA) stimulation by the lncRNA KCNQ1OT1 sponging miR-34c-5p promotes aerobic glycolysis in osteosarcoma to support metastasis (32).

Lactate dehydrogenase A (LDHA) catalyzes the conversion of pyruvate to lactate. The upregulation of LDHA is involved in cancer cell growth and migration, the development of stem-cell like traits, and chemoresistance (33). KDM6B regulates H3K27me3 demethylation in the promoter region of LDHA, thereby promoting LDHA expression and aerobic glycolysis in osteosarcoma cells, and hence facilitating tumor metastasis (34).

The m^6^A-reading protein YTH N^6^-methyladenosine RNA-binding protein 3 (YTHDF3) contributes to osteosarcoma progression by promoting aerobic glycolysis through enhancement of PGK1 mRNA stability in an m^6^A-dependent manner (35).

IDH1 is an important TCA cycle enzyme that catalyzes the conversion of isocitrate to α-ketoglutarate. High levels of IDH1 have been detected in osteosarcoma and correlated with poor survival. Hsp90-AHA1 was found to upregulate IHD1 and promote growth and metastasis in osteosarcoma (36).

Besides glucose metabolism, changes in lipid and amino acid metabolism have also been reported to participate in osteosarcoma metastasis. Lipid profiles differ in metastatic osteosarcoma cell lines compared to non-metastatic cells. For example, diacylglycerols are overexpressed in metastatic osteosarcoma cells, and the blockage of its synthesis can in fact inhibit cell migration (37). Highly metastatic osteosarcoma cell lines require glutamine for proliferation, and conversely, glutaminase-1 (GLS-1) inhibition limits metastatic progression in osteosarcoma (38).

CD47 is a key factor mediating immune evasion of tumor cells from the innate immune system. Increased uptake of leucine and glutamine in osteosarcoma cells through upregulation of LAT2 activates mTORC1 and subsequent c-Myc-mediated transcription of CD47, enabling evasion of innate immune mechanisms and thereby promoting metastasis (39).

#### Dysregulated pathways

**Figure 2:**
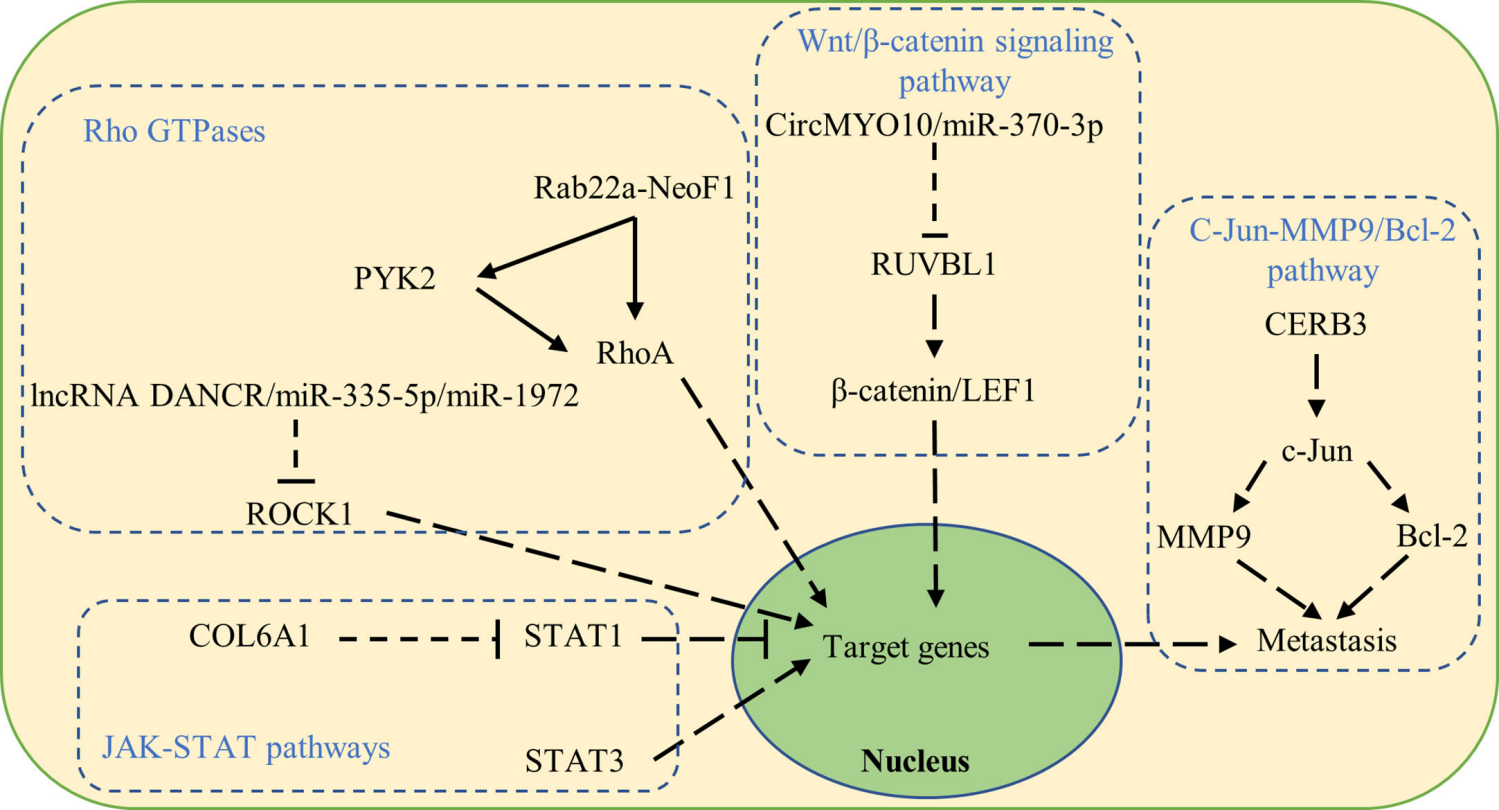
Signaling pathways that contribute to osteosarcoma metastasis when dysregulated.

Dysregulated signaling pathways have also been reported to be involved in osteosarcoma metastasis (**Figure 2**).

##### Wnt/β-catenin signaling pathway

The Wnt/β-catenin signaling pathway is reported to play a crucial role in cell fate determination, proliferation, and migration in cancer. Cytoplasmic β-catenin undergoes ubiquitination and proteasomal degradation mediated by a destruction complex composed of Axin, APC, PP2A, GSK3, and CK1α. On the other hand, nuclear translocated β-catenin acts as a transcriptional coactivator for the TCF/LEF family of transcription factors promoting the expression of Wnt-target genes such as C-myc, RUNX2, and CyclinD1, which subsequently promotes the epithelial–mesenchymal transition and facilitates osteosarcoma metastasis (40, 41).

RUVBL1 can be regulated by CircMYO10/miR-370-3p in osteosarcoma and influences osteosarcoma progression. Molecularly, RUVBL1 enhances the transcriptional activity of the β-catenin/LEF1 complex by mediating chromatin remodeling at the promoter regions of LEF1 target genes, consequently promoting osteosarcoma metastasis (42).

##### C-Jun-MMP9/Bcl-2 pathway

As upstream signaling agents of MMPs, mitogen-activated protein kinase (MAPK) is a family of serine/threonine kinases that includes extracellular signal-regulated kinase (ERK)1/2, c-Jun N-terminal kinase (JNK) 1/2, and p38. Activation of MAPK is followed by the phosphorylation of various cytosolic substrates that participate in numerous cellular activities such as cell proliferation, differentiation, invasion, migration, and death (43, 44).

Activated by CERB3, c-Jun upregulates MMP9 and Bcl-2 to promote osteosarcoma proliferation and metastasis (45).

##### Rho GTPases

Rho GTPases belong to the Ras superfamily of GTPases, which are implicated in cell proliferation, cell cycle progression, and migration. Dysregulation of Rho GTPase functions is involved in osteosarcoma progression and metastasis (46).

RhoA activation in tumor cells leads to osteosarcoma metastasis to the lung (47, 48). The fusion protein Rab22a-NeoF1 either directly binds and activates RhoA, or is secreted together with its binding partner PKY2 by tumor-positive cells, taken up by tumor-negative cells, and facilitating RhoA activation *via* PYK2 (3, 49).

The Rho-associated coiled-coil containing protein kinase 1 (ROCK1) was reported to be a proliferation- and metastasis-related gene in various cancers including osteosarcoma (50). ROCK1 is regulated in osteosarcoma by lncRNA DANCR/miR-335-5p/miR-1972 (51).

##### JAK-STAT pathways

Signal transducer and activator of transcription (STAT) consists of seven members involved in the regulation of cell proliferation, differentiation, and survival. The activation of STAT1 in osteosarcoma cells suppressed EMT, resulting in increased apoptosis and cell cycle arrest, and decreased colony formation, cell migration, and invasion. Increased expression of COL6A1 promoted STAT1 degradation, which subsequently facilitated osteosarcoma metastasis (52). Furthermore, STAT3 is overexpressed in osteosarcoma and associated with poor survival. Activation of STAT3 upregulates the expression of target oncogenes and facilitates osteosarcoma metastasis (53).

#### Transcription factors

Dysregulation of transcription factors also contributes to osteosarcoma metastasis. NRF2 regulates intracellular ROS balance, the AMPK/mTOR autophagy signaling pathway, and the Warburg effect. TRIM22 inhibits osteosarcoma progression by binding to and destabilizing NRF2 in a KEAP1-independent manner (54).

RUNX proteins are DNA-binding transcription factors that regulate the expression of multiple genes involved in cellular differentiation and cell-cycle progression. RUNX2 is essential to osteoblast maturation and bone development, and can either suppress or promote carcinogenesis based on the clinical condition (55). Studies of osteosarcoma tumors have revealed that levels of RUNX2 DNA, RNA, and protein are significantly elevated in osteosarcoma tumors. Chromobox homolog4 (CBX4) is overexpressed in osteosarcoma cell lines and tissues, and promotes osteosarcoma metastasis by transcriptionally upregulating RUNX2 *via* the recruitment of GCN5 to the RUNX2 promoter (56).

Cyclic AMP-responsive element-binding protein 3 (CERB3), also known as LZIP or LUMAN, is a member of the leucine zipper transcription factor family. Its tumor-promoting role in osteosarcoma is regulated by circular RNA circTADA2A-miR-203a-3p. Molecularly, CREB3 can bind directly to the c-Jun promoter and regulate the transcriptional activity of c-Jun in osteosarcoma. MMP9 and Bcl-2 can be regulated by c-Jun and participate in CREB3-c-Jun modulated osteosarcoma progression (45).

The transcription activators YAP/YAZ regulate EMT through AXL in osteosarcoma and influences cell differentiation, cell fate, and metastasis (57).

### Tumor microenvironment

The tumor microenvironment includes cellular components, extracellular matrix, vesicles, and secreted molecules that interact with each other to regulate tumor progression, immune evasion, drug resistance, and metastasis (58, 59).

The cellular components of the tumor microenvironment are mainly composed of infiltrating lymphocytes, macrophages, fibroblasts, and platelets. The composition and functions of these cells are dynamically regulated by local tumor cells and can be influenced by therapeutic agents. The recruitment and/or activation of certain cells in the microenvironment play pivotal roles in osteosarcoma metastasis (60).

The prognostic role of tumor-infiltrating lymphocytes in the osteosarcoma tumor microenvironment has been explored. The presence of tumor-infiltrating CD4+ or CD8+ cells was correlated with improved overall survival and progression-free survival in osteosarcoma patients (61).

In addition to tumor-infiltrating lymphocytes, the functional states of macrophages in the tumor microenvironment have also been associated with osteosarcoma progression and metastasis. M1-polarized macrophages are generally regarded as tumor-suppressing, while M2-type macrophages exhibit tumor-promoting roles in osteosarcoma. Molecularly, M2-type macrophages secrete cytokines such as IL-10, TGF-β, and VEGF to promote osteosarcoma EMT and metastasis (62). The M2-polarized macrophages are primarily induced by the activation of Stat3 secondary to stimulation by tumor cell secretions such as exosomes or vesicles. For instance, tumor-derived exosomes have been reported to induce M2 macrophage polarization *via* Tim-3 to promote osteosarcoma metastasis (62). PYK2 secretion by osteosarcoma cells recruits bone marrow-derived cells (BMDCs) and induces M2 macrophage polarization by activating Stat3 in macrophage cells (8). In the presence of chemotherapy, macrophages secrete IL-18 and enable the upregulation of LAT2 in adjacent osteosarcoma cells, which, in turn, promotes tumor evasion by upregulating CD47 (39).

Cancer-associated fibroblasts (CAFs) comprise a large proportion of cells in the tumor microenvironment. These cells can be identified by the presence of α-smooth muscle actin, fibroblast activation protein, and vimentin. Activated CAFs are thought to promote tumor cell growth, invasion, metastasis, drug resistance, and reprogramming (63). At a molecular level, CAFs build up and remodel the extracellular matrix, enabling tumor cells to invade through the TME. In addition, CAFs modulate cancer cell behavior through the secretion of growth factors, cytokines, and chemokines such as IL-1beta, IL-6, IL-8, TGF-β, and collagen (63).

CAFs can be activated and reprogrammed by various mechanisms, contributing to tumor metastasis. Increased levels of COL6A1 in tumor cells are packaged into exosomes and transported to activated CAFs, which, in turn, promote tumor invasion and metastasis by secreting TGF-β (52). CAFs in the lung can also be reprogrammed to support osteosarcoma metastasis under the influence of TGF-β1 found in osteosarcoma-derived extracellular vesicles (64).

Platelet aggregation and activation can be induced by tumor cells to support tumor metastasis in osteosarcoma. Osteosarcoma cells highly express PDPN, which binds with CLEC-2 on the surface of platelets, leading to platelet activation and subsequent tumor metastasis. At a molecular level, activated platelets secrete various growth factors and cytokines such as PDGF, TGF-β, and LPA, thereby inducing EMT and promoting cell migration and invasion in osteosarcoma. In addition, aggregated platelets form clusters with tumor cells, which are then trapped in the microvasculature of various organs such as the lung, triggering tumor metastasis (65).

The extracellular matrix (ECM) is extensively altered in osteosarcoma, beginning with the collagens and proteoglycans that make it up. Increased expression of several sarcomatous matrix proteins has been associated with poor response to chemotherapy and poor prognosis in clinical studies of osteosarcoma. NELL1 is a secreted osteoinductive protein, which has bone anabolic and anti-osteoclastic effects. NELL1 can promote osteosarcoma metastasis by regulating the expression of key matricellular proteins through the induction of FAK/Src signaling (66).

The procollagen C-proteinase enhancer protein (PCOLCE) is a secreted glycoprotein that enhances procollagen C-proteinase participation in ECM reconstruction. PCOLCE is upregulated by TWIST1 in osteosarcoma and promotes osteosarcoma metastasis to the lung (67).

The extracellular matrix glycoprotein tenascin-C is highly expressed in the tumor microenvironment and promotes the migration, invasion, and metastatic progression of osteosarcoma. Tenascin-C functions by binding with its receptor integrin α9β1, which abolishes actin stress fiber formation and inhibits YAP and its downstream target gene expression (68).

Extracellular vesicles (EVs) are secreted by both tumor cells and their adjacent non-tumor counterparts with diameters ranging from 30 to 150 nm (69). These vesicles are rich in biologically active components such as proteins, lipids, and nucleic acids, and play important roles in the exchange of biomolecules between different cell types (70). Many studies have correlated EVs with carcinogenesis, progression, and metastasis in osteosarcoma (71–73).

### Results of the recent clinical trials of advanced or metastatic osteosarcoma

To date, there remains no established effective treatment for metastatic osteosarcoma. Multiple clinical trials have been conducted in recent years to investigate the viability of novel agents or treatment combinations. We compiled key findings from clinical trials in advanced or metastatic osteosarcoma within the last 7 years (summarized in **Table 1**).

**Table 1:** Results of recent clinical trials involving patients with advanced or metastatic osteosarcoma.

Treatment type	Intervention agents	Inclusion criteria	Trial phase	Number of cases	Study design	Results	Year of publication	References
Chemotherapy	Pegylated liposomal doxorubicin+cisplatin	Metastatic and recurrent osteosarcoma	Phase 1	15	Single arm, multiple center	6-week ORR, 13.3%; DCR, 66.7%	2022	Xi-zhi Wen (74)
Targeted therapy	Sorafenib and everolimus	Relapsed or unresectable osteosarcoma progressing after standard treatment (methotrexate, cisplatin, and doxorubicin, with or without ifosfamide)	Phase 2	38	Single arm, multiple center	6-month PFS, 45%	2015	Giovanni Grignani (75)
	Robatumumab	Resectable osteosarcoma metastases (Group 1, *n* = 31), Unresectable osteosarcoma metastases (Group 2, *n* = 29)	Phase 2	60	Case–control study	>6-month DCR, 9.7% *vs*. 0; median OS 24 m *vs*. 8.2 m	2016	Peter M. Anderson (76)
	Regorafenib *vs*. placebo	Progressive metastatic osteosarcoma	Phase 2	42	Randomized double-blind, multi center	Median PFS 3.6 m *vs*. 1.7 m	2019	Lara E. Davis (77)
	Regorafenib *vs*. placebo	Metastatic osteosarcoma	Phase 2	43	Randomized double-blind, multi center	8-week PFS 65% *vs*. 0	2019	Florence Duffaud (78)
	Dinutuximab	Recurrent pulmonary osteosarcoma in complete surgical remission	Phase 2	39	Single arm, single center	12-month DCR (event-free survival), 28.2%	2022	Pooja Hingorani (79)
Chemotherapy and targeted therapy	Pazopanib+topotecan	Metastatic or unresectable osteosarcoma	Phase 2	28	Single arm, open	12-w PFS, 69.5%; 24-w PFS, 45.4%; 12-month PFS, 18.2%;median PFS, 4.5 months; median OS, 11.1 months; ORR, 4%.	2021	Brian Schulte (80)
Immunotherapy	Trivalent ganglioside vaccine+ OPT-821 *VS* OPT-821	Metastatic osteosarcoma following complete metastasectomy as subgroup	Phase 2	14	Randomized double-blind, multi center	12-month RFS 34.5% *vs*. 34.8% in general, subgroup data not shown	2022	Evan Rosenbaum (81)
	Pembrolizumab	Advanced or metastatic osteosarcoma	Phase 2	22	Single arm, multiple center	BOR, 5%	2017	Hussein A Tawbi (82)
	Ipilimumab	Advanced or metastatic osteosarcoma	Phase 1	8	Single arm, multiple center	6-w PFS, 0%	2016	Melinda S. Merchant (83)
Chemotherapy and immunotherapy	Chemotherapy(4 agents) and interleukin-2	Primary metastatic osteosarcoma	Phase 2	35	Single arm, single center	3-y EFS, 34.3%; 3-y OS, 45.0%	2017	Cristina Meazza (84)
Targeted therapy and immunotherapy	Nivolumab+bempegaldesleukin	Advanced or metastatic osteosarcoma	Phase 2	10	Single arm, open	6-month DCR, 0%; median PFS, 2 months; median OS, 6.3 months	2022	Sandra P. D’Angelo (85)
	Durvalumab plus tremelimumab	Advanced or metastatic osteosarcoma	Phase 2	5	Single arm, single center	12-w PFS, 0%	2022	Neeta Somaiah (86)
Radiotherapy	Radium 223	Recurrent/metastatic osteosarcoma	Phase 1	18	Single arm, multiple center	Median OS, 25 w	2019	Vivek Subbiah (87)

ORR, objective response rate; PFS, progression free survival; DCR, disease control rate; OS: overall survival; RFS, recurrent free survival; BOR, best of response; EFS, event-free survival.

Wen et al. reported a Phase 1 clinical trial investigating the efficacy of the combination therapy of pegylated liposomal doxorubicin and cisplatin in metastatic and recurrent osteosarcoma (74). In 15 cases, the 6-week objective response rate was 13.3% and the disease control rate was 66.7%. Other trials on targeted therapies such as regorafenib (77, 78), dinutuximab (79), robatumumab (76), sorafenib, and everolimus (75) demonstrated limited success with the overall 6-month progression-free survival rate of less than 50%.

The efficacy of combinatorial chemotherapy and targeted therapy treatments has also been tested in metastatic or unresectable osteosarcoma. A single-arm Phase 2 clinical trial involving 28 patients treated with pazopanib and topotecan failed to show any significant improvement in survival (6-month progression-free survival of 45.4%) (80).

Immunotherapy is an emerging treatment modality that has shown promising results in selected cases in melanoma and lung cancers. However, osteosarcoma patients did not seem to respond well to immune checkpoint inhibitors (81–83). The addition of interleukin-2 immunotherapy to a four-agent chemotherapy regimen for treating metastatic osteosarcoma did result in a 3-year event-free survival of 34.3% and 3-year overall survival of 45.0% (84), but a combination of targeted therapy and immunotherapy did not elicit better outcomes (85, 86).

Radiotherapy with radium 223 was also assessed in a clinical trial that involved 18 patients with recurrent or metastatic osteosarcoma (87). This Phase 1 single-arm multi-center trial reported a median overall survival of 25 weeks.

### Ongoing clinical trials

There are currently several ongoing clinical trials involving metastatic osteosarcoma registered in ClinicalTrials.gov (**Table 2**). These include Phase 1, 2, and 3 trials. Interventions being investigated include chemotherapy, immunotherapy, radiotherapy, or targeted therapy alone; and combinatorial chemotherapy and immunotherapy, chemotherapy and targeted therapy, and targeted therapy and immunotherapy. Favorable outcomes from these trials have the potential to transform the landscape of clinical management of metastatic osteosarcoma.

**Table 2:** Ongoing clinical trials involving patients with metastatic osteosarcoma.

Intervention type	Interventions	Phases	Enrollment	Status	NCT Number
Chemotherapy	Methotrexate, Doxorubicin, Cisplatin, Ifosfamide, and Etoposide (MAPIE) with or without Zoledronic acid	Phase 3	318	Active, not recruiting	NCT00470223
	Drug: Ascorbate	Early Phase 1	20	Recruiting	NCT04634227
	Drug: Doxorubicin	Phase 1	11	Active, not recruiting	NCT02811523
Chemotherapy+immunotherapy	Drug: Mifamurtide|Combination Product: EI or M-API regimen depending on patient age	Phase 2	126	Recruiting	NCT03643133
	Drug: Cyclophosphamide|Drug: attIL2-T cells	Phase 1	40	Not yet recruiting	NCT05621668
	Genetic: GD2 T cells|Biological: VZV vaccine|Drug: Fludarabine|Drug: Cyclophosphamide	Phase 1	26	Active, not recruiting	NCT01953900
Chemotherapy+targeted therapy	Drug: Chemotherapy (gemcitabine and docetaxel) plus BIO-11006	Phase 2	10	Active, not recruiting	NCT04183062
	Methotrexate, Doxorubicin, and Cisplatin (MAP) with or without Cabozantinib	Phase 2|Phase 3	1,122	Not yet recruiting	NCT05691478
	Drug: Apatinib|Drug: GD regimen	Phase 2	43	Active, not recruiting	NCT03742193
	Drug: carboplatin|Drug: dasatinib|Drug: etoposide phosphate|Drug: ifosfamide	Phase 1|Phase 2	7	Active, not recruiting	NCT00788125
Immunotherapy	Biological: Dinutuximab|Biological: Sargramostim	Phase 2	41	Active, not recruiting	NCT02484443
	Biological: Denosumab	Phase 2	56	Active, not recruiting	NCT02470091
	Biological: Durvalumab|Biological: Oleclumab	Phase 2	75	Recruiting	NCT04668300
	Biological: Aerosolized Aldesleukin	Phase 1	70	Active, not recruiting	NCT01590069
	Drug: Iscador*P	Phase 2	32	Not yet recruiting	NCT05726383
	Biological: Ipilimumab|Biological: Nivolumab|Other: Quality-of-Life Assessment	Phase 2	164	Active, not recruiting	NCT02500797
Radiotherapy	Drug: Iodine I 131 MOAB 8H9	Phase 1	120	Active, not recruiting	NCT00089245
Targeted thearpy+immunotherapy	Biological: Atezolizumab|Drug: Cabozantinib	Phase 2	40	Not yet recruiting	NCT05019703
	Drug: Regorafenib 40 MG|Drug: Regorafenib 20MG|Drug: Nivolumab	Phase 2	48	Recruiting	NCT04803877
Targeted therapy	Drug: Natalizumab	Phase 1|Phase 2	20	Recruiting	NCT03811886
	Drug: Cabozantinib S-malate	Phase 2	90	Active, not recruiting	NCT02243605
	Drug: Regorafenib|Drug: Placebo	Phase 2	132	Recruiting	NCT02389244

## Discussion

Osteosarcoma is the most common primary bone malignancy affecting children and young adults. More than 10% of patients are diagnosed with distant metastasis, and the 5-year overall survival of these patients is approximately 20%. However, current management options to prevent metastasis are limited and ineffective.

### Emerging treatment options

**Figure 3:**
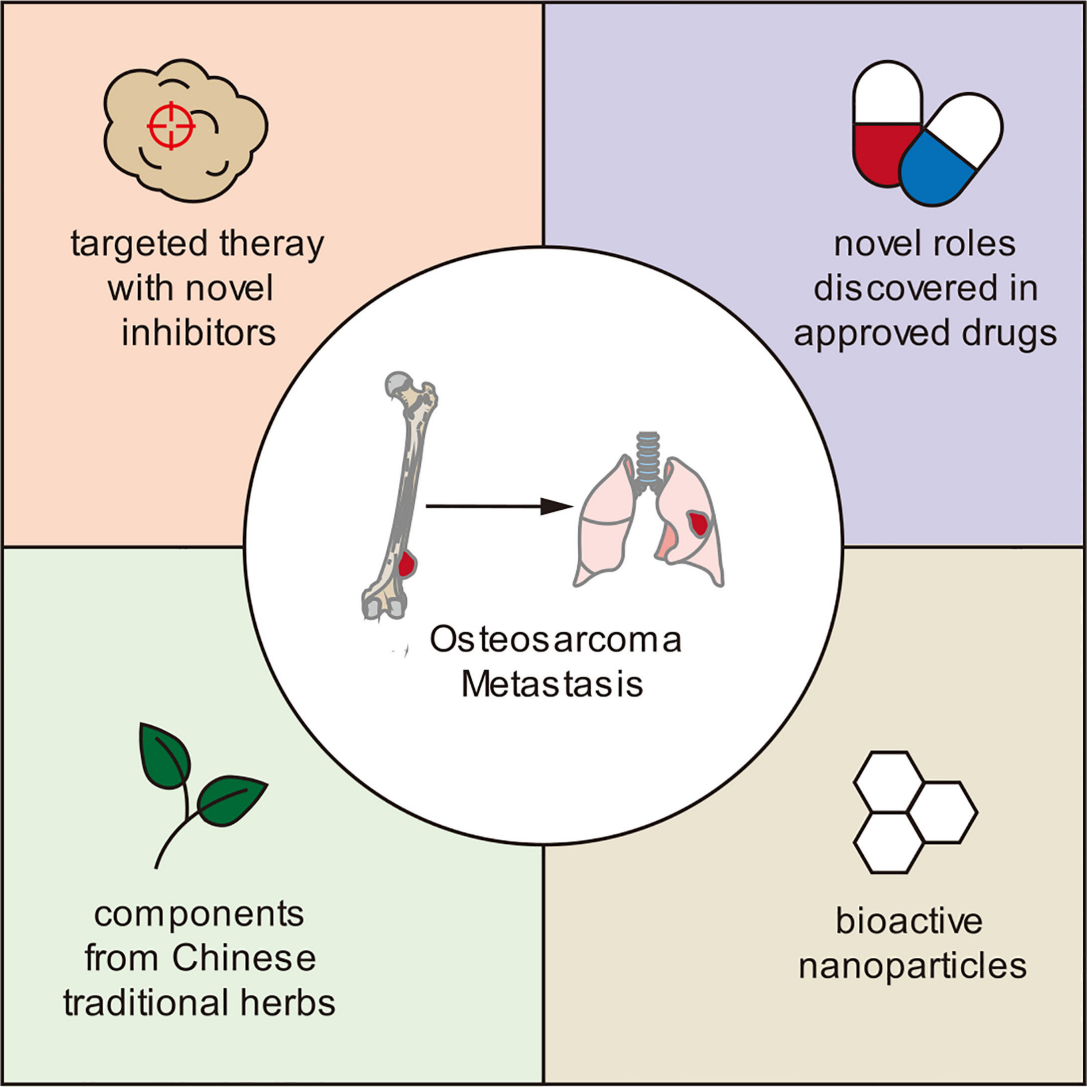
Novel therapies that have demonstrated anti-metastatic effects in osteosarcoma in preclinical studies.

Growing research on tumor cell alterations, behavior, and their surrounding microenvironment has informed the investigation of novel treatment options in preclinical settings. These include inhibitors targeting key metastasis-promoting proteins, approved drugs with newly discovered anti-metastatic roles, bioactive nanoparticles, and traditional Chinese medicine agents (**Figure 3**).

#### Inhibitors targeting key metastasis-promoting proteins

Multiple key drivers of osteosarcoma metastasis have been reported, and inhibitors targeting these specific drivers have been developed and assessed. The covalent CDK7 inhibitor THZ2 demonstrated significant suppression of osteosarcoma tumor growth and metastasis by targeting super-enhancer-associated oncogenes (88). Tegavivint, a novel β-catenin/transducing β-like protein 1 (TBL1) inhibitor, exhibits anti-proliferative activity against osteosarcoma cells *in vitro* and *in vivo* through downregulation of the Wnt signaling pathway (89). CDK12 has been reported to facilitate genome stability through the regulation of DDR genes; accordingly, the CDK12 inhibitors THZ531 and E9 were found to disrupt osteosarcoma metastasis (90). BMTP-11 targets IL-11R α and inhibits osteosarcoma tumor growth and lung metastasis (91). A quinoline-based DNA methyltransferase inhibitor can induce cell cycle arrest and osteoblastic differentiation in osteosarcoma. It also showed synergistic effects with doxorubicin and cisplatin in treating osteosarcoma (92).

#### Approved drugs with newly discovered anti-metastatic roles

Drugs previously FDA-approved for other indications have been reported to inhibit osteosarcoma progression and metastasis. The FDA-approved DNA methylation inhibitor decitabine has demonstrated the ability to decrease proliferation, induce osteoblast differentiation, and reduce metastasis to visceral organs. Decitabine exposure in osteosarcoma reduces the protein expression of the metastasis-associated markers VIMENTIN, SLUG, ZEB1, and MMP9, with a concurrent decrease in mRNA expression of the known stem cell markers SOX2, OCT4, and NANOG. Normal osteoblasts express estrogen receptor α (ERα), whereas osteosarcoma cells do not due to promoter DNA methylation. Treatment of 143B osteosarcoma cells with decitabine led to ERα expression and decreased proliferation and induction of osteoblast differentiation (93).

Pramlintide, an FDA-approved drug for type 2 diabetes, was found to inhibit glycolysis and osteosarcoma tumor growth both *in vitro* and *in vivo* by inducing apoptosis (94). Melatonin attenuates chemokine CCL24 levels through inhibition of the JNK pathway to hinder human osteosarcoma cell invasion (95). All-trans retinoic acid prevents osteosarcoma metastasis by inhibiting M2 polarization of tumor-associated macrophages (96).

#### Bioactive nanoparticles

Bioactive nanoparticles (NPs), such as gold NPs, copper oxide NPs, iron oxide NPs, and zinc oxide nanoparticles (ZnO NPs), have been recently discovered to possess significant tumor-suppressing roles (97–99). ZnO NPs can inhibit osteosarcoma metastasis by degrading β-catenin in the HIF-1 α/BNIP3/LC3B-mediated mitophagy pathway (100).

#### Traditional Chinese medicine agents

The anti-tumor roles of traditional Chinese medicines and herbs have been explored in osteosarcoma. Ailanthone (AIL), a major component of the Chinese medicine *Ailanthus altissima*, can induce metabolic reprogramming in osteosarcoma, leading to growth inhibition both *in vitro* and *in vivo*. Molecularly, AIL induces cell cycle arrest and apoptosis in osteosarcoma cells by downregulating the serine biosynthetic pathway (101). Other natural compounds or herbs such as degalactotigonin (102) and shikonin (103) have also been reported to inhibit osteosarcoma growth and metastasis.

### Navigating the challenges of osteosarcoma

**Figure 4:**
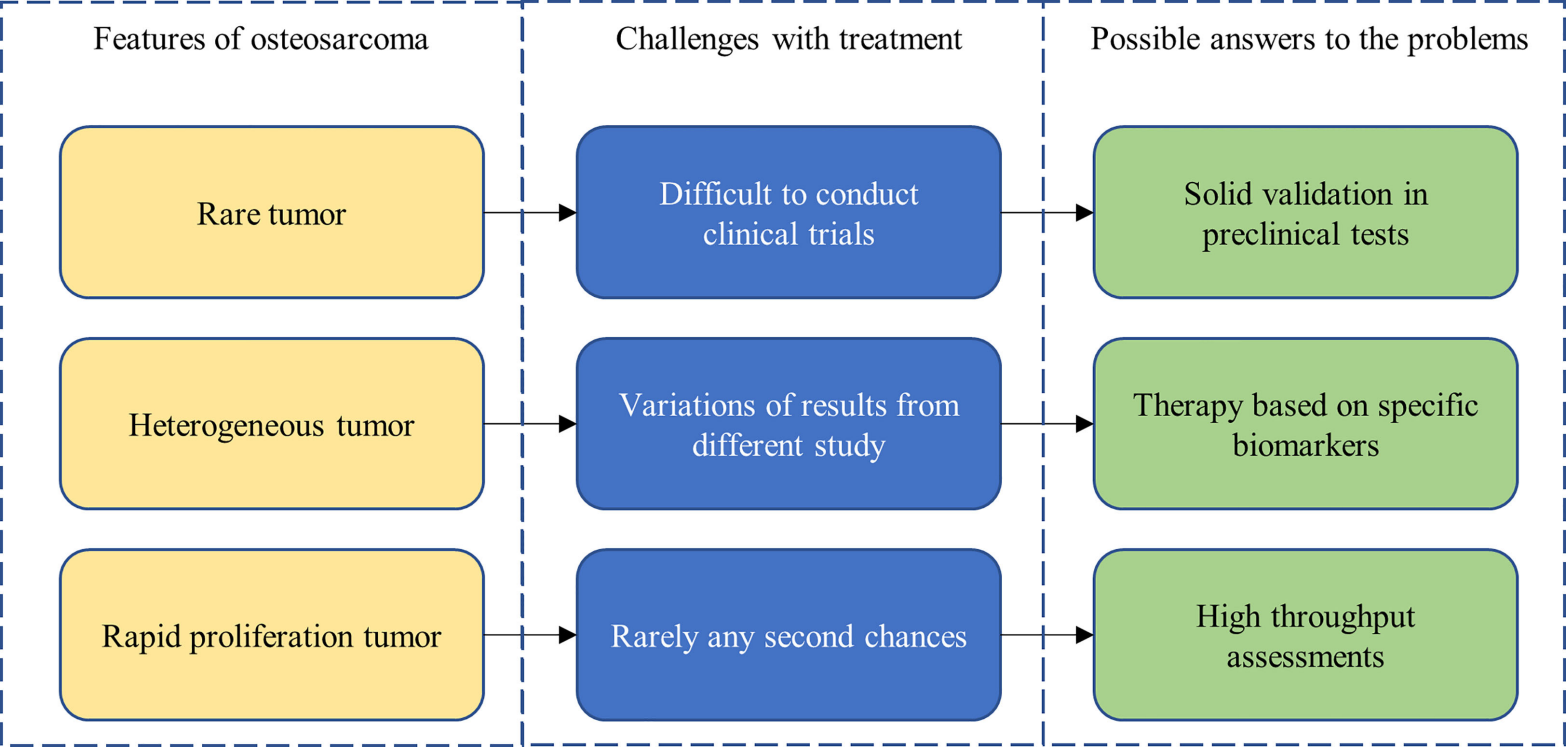
Features of, treatment challenges in, and potential solutions for osteosarcoma.

Osteosarcoma research is particularly challenging (**Figure 4**). The low prevalence of osteosarcoma makes the conducting of rigorous clinical trials especially challenging. Heterogeneity within and between patient tumors also limits the generalizability of study findings. Thankfully, advancements in biotechnology and molecular techniques have paved the way for solutions to some of these challenges. For example, patient-derived xenograft models and organoid cultures have emerged as viable cancer models for experimentation, offering increased biomimicry, which should lead to stronger correlations with patient outcomes. Furthermore, detailed molecular characterization of osteosarcoma has allowed for the development of personalized therapies that target specific biomarkers and patient genomic profiles, increasing efficacy of potential treatments.

## Conclusion

We reviewed the current literature on contributors to osteosarcoma metastasis, including genomic and epigenomic changes, metabolic reprogramming, transcription factors, dysregulation of physiologic pathways, and alterations to the tumor microenvironment. In addition, we discussed potential emerging therapies to suppress osteosarcoma metastasis. Further research on the molecular mechanisms of osteosarcoma metastasis, combined with growing molecular technologies, can inform the development of novel, personalized, and targeted therapies to ultimately improve outcomes in osteosarcoma patients.

## Author contributions

Conceptualization, XD and HW. Writing—original draft preparation, XD. Writing—review and editing, BZ, LP and RZ. Visualization, HW. Supervision, WY. Funding acquisition, XD and HW. All authors contributed to the article and approved the submitted version.
